# *BBS4* Is Necessary for Ciliary Localization of TrkB Receptor and Activation by BDNF

**DOI:** 10.1371/journal.pone.0098687

**Published:** 2014-05-27

**Authors:** Carmen C. Leitch, Norann A. Zaghloul

**Affiliations:** Division of Endocrinology, Diabetes, and Nutrition, University of Maryland School of Medicine, Baltimore, Maryland, United States of America; University of Louisville, United States of America

## Abstract

Primary cilia regulate an expanding list of signaling pathways in many different cell types. It is likely that identification of the full catalog of pathways associated with cilia will be necessary to fully understand their role in regulation of signaling and the implications for diseases associated with their dysfunction, ciliopathies. Bardet-Biedl Syndrome (BBS) is one such ciliopathy which is characterized by a spectrum of phenotypes. These include neural defects such as impaired cognitive development, centrally mediated hyperphagia and peripheral sensory defects. Here we investigate potential defects in a signaling pathway associated with neuronal function, brain derived neurotrophic factor (BDNF) signaling. Upon loss of *BBS4* expression in cultured cells, we observed decreased phosphorylation and activation by BDNF of its target receptor, TrkB. Assessment of ciliary localization revealed that, TrkB localized to the axonemes or basal bodies of cilia only in the presence of BDNF. Axonemal localization, specifically, was abrogated with loss of *BBS4*. Finally, we present evidence that loss of the ciliary axoneme through depletion of *KIF3A* impedes activation of TrkB. Taken together, these data suggest the possibility of a previously uninvestigated pathway associated with perturbation of ciliary proteins.

## Introduction

Bardet-Biedl Syndrome (BBS) is a pleiotropic disorder characterized by phenotypes including retinal degeneration, polydactyly, hypogonadism and renal defects [1]. Among the most highly penetrant features are deficits in brain function, such as intellectual disability and impaired hypothalamic regulation of satiety, suggesting that the 18 known BBS genes [2], [3], [4], [5], [6] may play an important role in the regulation of pathways associated with these traits. By virtue of BBS protein localization at the basal body of primary cilia and their role in trafficking of ciliary cargo [7], [8], BBS is part of the group of disorders known as ciliopathies. This, compounded with the role of primary cilia as a central hub for regulation of various signaling pathways [9], suggests that the intracellular regulation of neuronal cues may be dependent on primary cilia.

Primary cilia have been implicated in the proper transduction of multiple signaling pathways. These include pathways necessary for development and differentiation, such as Sonic hedgehog (Shh) and Wnt, [10], [11], as well as those necessary for regulation of response to physiological cues, such as insulin [12] and melanin-concentrating hormone [13]. Additionally, neuronal signaling receptors, including several neuropeptide receptors have been localized to cilia and require interaction with ciliary proteins. This includes the regulation of leptin receptor trafficking by BBS proteins [14]. Likewise, neuropeptide Y receptors are trafficked by BBS proteins to neuronal cilia [15]. These studies and others suggest important roles for BBS proteins in receptor trafficking to primary cilia in neurons. The extent to which this trafficking is necessary for other neuronal signaling, however, is unclear.

Brain derived neurotrophic factor (BDNF) signaling is a secreted neurotrophin that is necessary for neuronal development and synaptic plasticity. It also plays a pivotal role in regulation of satiety and weight [16]. It binds one of two receptors, the tropomyosin receptor kinase family receptor tyrosine kinase, TrkB, and the p75 neurotrophin receptor. Loss of BDNF-mediated activation of TrkB results in phenotypes reminiscent of BBS, included hyperphagia-driven obesity [17], [18], [19]. In light of this, we hypothesized that there may be an intracellular link between BBS proteins, cilia, and BDNF signaling. Therefore, we assessed the localization and activation of TrkB receptor by BDNF in cultured cells depleted of *BBS4*. Here, we present evidence of a role for *BBS4* in mediating the phosphorylation of TrkB by BDNF. We also present evidence implicating the proper localization of TrkB to the ciliary axoneme in this activation. Taken together, these data suggest the possibility of a previously unexplored signaling mechanism for neuronal phenotypes associated with BBS.

## Materials and Methods

### Cell culture & Transfections

hTERT-RPE1 (ATCC) and SH-SY5Y cells (Kindly received from I. Goldberg, University of Maryland) were cultured in DMEM:F:12 with 10% FBS/0.1% hygromycin or DMEM:F:12 with 10% FBS/1% Non-essential Amino Acids/1%Pen-Strep, respectively. SH-SY5Y cells were treated with retinoic acid to a final concentration of 10 µM in culture medium (Sigma-Aldrich R2625) the day after plating for 5 days prior to transfection. For transfection, cells in growth media were plated on coverslips or in culture dishes and transfected at 85–90% confluency using Lipofectamine 2000 (Life Technologies) according to manufacturer's protocol with the following constructs at 4 µg/2.0 mL: shBBS4 in pSUPER (Gerdes et al. 2007); shBBS4-3′UTR, shKIF3A and shKIF3A-3′UTR in pLKO.1-puro (Sigma-Aldrich MISSION collection); *BBS4* and *KIF3A* ORFs cloned into pCS2+. Transfection with empty vector (pSUPER, pLKO.1-puro, or pCS2+) was used as control. Transfection efficiency was determined for all constructs in both cell types by co-transfection with a GFP-expressing plasmid and cells were used for further analysis after ensuring a transfection efficiency of 85–95% of cells. BDNF (eBioscience 14–8365) diluted in culture media to 50 ng/ml was added to cells 48 hours (hTERT-RPE1) or 72 hours (SH-SY5Y) post-transfection and remained on cells for 24 hours or 15 minutes, respectively, unless otherwise indicated.

### Western Blot and Quantification

Cells were washed in ice-cold PBS and harvested in ice-cold buffer containing 50 mM Tris, 150 mM MgCl_2_, 1% NP-40, protease inhibitor (Sigma) and phosphatase inhibitor (Sigma). Cells were incubated in lysis buffer on ice for 15 minutes, vortexing every 5 minutes. Lysates were centrifuged in an Eppendorf 5415R at 4°C, 8000xg for 10 minutes. Supernatant was collected and incubated 1∶1 in Laemmli sample buffer (BioRad) plus β-mercaptoethanol for 10minutes and boiled for 5 minutes. Samples were run on a NuPage 4–12% Bis-Tris gel (Life Technologies) with MOPS running buffer (Life Technologies). Proteins were transferred onto a nitrocellulose membrane at 30 V for 90 minutes on ice. Membranes were blocked in 5% milk in TBST for one hour at room temperature with rocking. Membranes were incubated overnight in primary antibody, washed the following day in TBST and incubated in the appropriate secondary antibody. Blots were developed using ECL substrate (Pierce) using a FluorChemQ system and AlphaView Software. Blots were probed for detection of a TRKB band at approximately 95 kDa MW and subsequently stripped for detection of phosphorylated TRKB (pTRKB), also near 95 kDa, followed by stripping for Actin loading control (42 kDa) using ReStore Western Blot Stripping Buffer (Thermo Scientific). The following primary antibodies and concentrations were used: Anti-TRKB (BD Transduction Laboratories 610101, 1∶1000), Anti-phosphoNTRK2/pTRKB (Y515; Sigma-Aldrich SAB4503785, 1∶1000 or Y705; Abcam ab52191, 1∶1000), Anti-ACTIN (Sigma-Aldrich A2103, 1∶1500). The following secondary antibodies and concentrations were used: Anti-mouse IgG HRP-linked (Cell Signaling Technology 7076, 1∶2500), Anti-rabbit IgG HRP-linked (Cell Signaling Technology 7074, 1∶2500). Western blots were quantified by densitometry analysis using ImageJ software and quantification of each band relative to ACTIN. TRKB activation was measured as the ratio of pTRKB protein to TRKB protein for each lane. Average activation was calculated from a minimum of 3 (and up to 5) separate experiments.

### Immunofluorescence and Quantification

Cells plated on coverslips and transfected were fixed at 100% confluency in ice cold 1∶1 methanol:acetone for 2 minutes followed by two 1XPBS washes. Double immunostaining was carried out starting with a one-hour PBS/10% serum/1% BSA block, one-hour primary antibody incubation at room temperature, second primary antibody incubation for one-hour at room temperature, and a one-hour secondary antibody incubation, with both secondary antibodies mixed together. The following primary antibodies were used: anti-TRKB (BD Transduction Laboratories 610101 1∶1000), anti-pTRKB (Abcam ab52191, 1∶1000), anti-ARL13B (Proteintech 17711-1-AP, 1∶1000), anti-γ-tubulin (Sigma-Aldrich T5192, 1∶1000. Species-specific secondary antibodies (AlexaFluor, Life Technologies) were used at 1∶1000. Following primary and secondary antibody incubations, coverslips were washed in 1XPBS, incubated in DAPI (0.2 µg/ml in PBS), mounted in Prolong Gold Antifade (Life Technologies), and imaged at 100× magnification with an Olympus IX50 with cellSens imaging software using deconvolution. Co-localization was quantified by assessing overlap of fluorescence in a single focal plane between ciliary markers and TRKB or pTRKB and counting the proportion of either basal bodies or axonemes that co-localize in 75–100 cells across a minimum of 3 separate experiments. Measurement of cilia length was calculated using ImageJ software on imaged hTERT-RPE1 cells labeled with antibodies against ARL13B.

### Quantitative RT-PCR

RNA was extracted from cells using Trizol reagent (Life Technologies) according to manufacturer's protocol and purified using the RNeasy Kit (QIAGEN). cDNA was transcribed using the Fermentas First Strand cDNA Transcription Kit (Thermo Scientific) according to manufacturer's protocol, diluted to 1∶9 (all genes but reference) or 1∶90 (reference gene only) and added to a reaction including target-specific primers (sequences available upon request) and LightCycler 480 SybrGreen (Roche) and run on a LightCycler 480 (Roche) for 5 minutes at 95°C then 40 cycles of 95°C (10 s), 58°C (15 s), 72°C (10 s) then 5 minutes at 72°C. A reverse-transcriptase-free sample was used as a negative control. All samples were run in duplicate with the C_T_ value normalized to GAPDH to calculate relative expression for each gene in each sample. Biological replicates were repeated a minimum of 3 times per treatment. Primer sets used were designed to detect the longest transcript isoforms of BDNF (NM_170735.5) and human NTRK2/TRKB (NM_006180.3). Primer sequences available upon request.

## Results

### Activation of TRKB is reduced in BBS4-depleted cells

**Figure 1:**
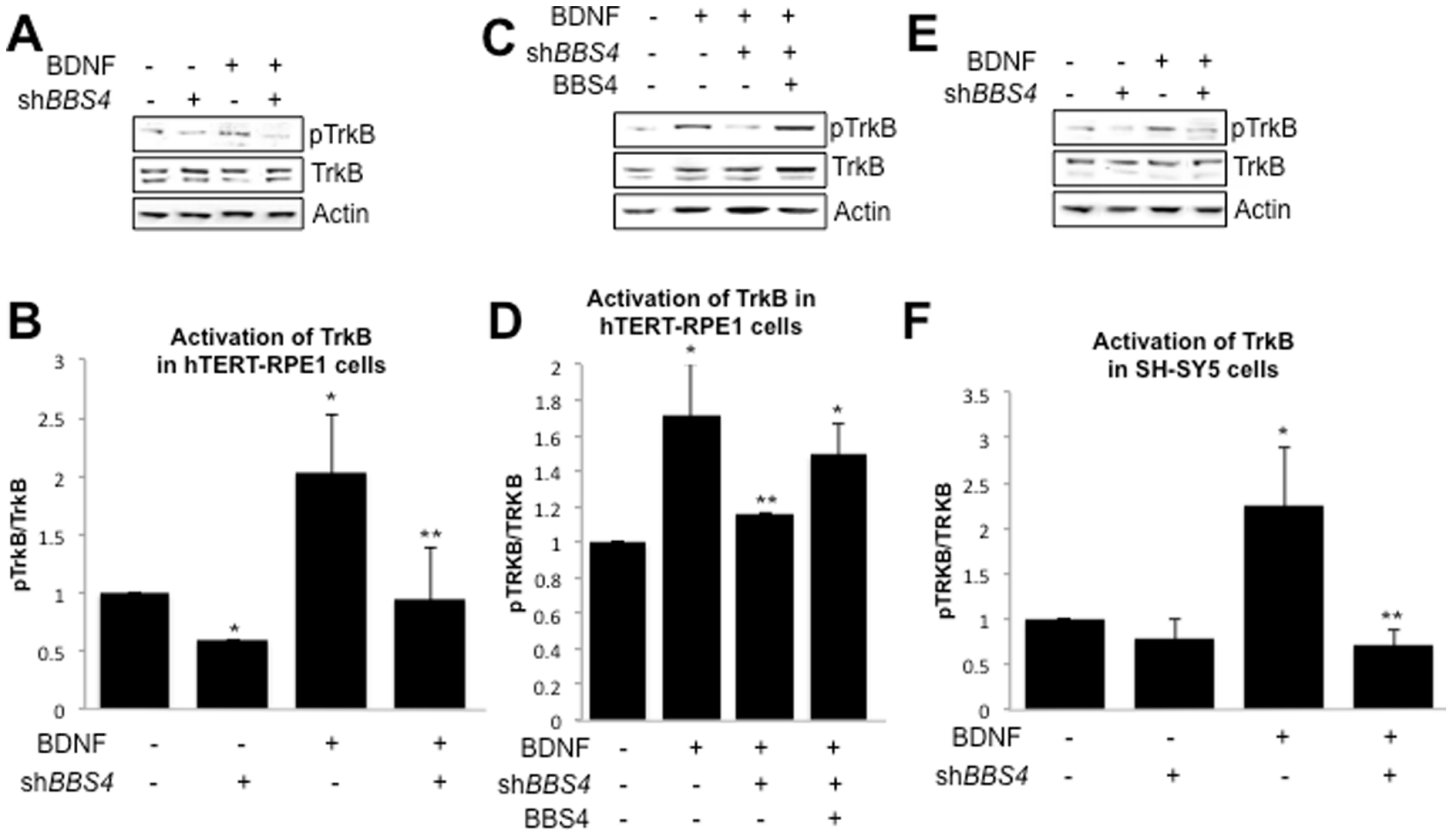
BDNF-induced phosphorylation of TRKB is reduced in *BBS4*-deficient cells. (A) Western blot detection in hTERT-RPE1 cells of phosphorylated TRKB (pTRKB) and unphosphorylated TRKB, as well as Actin as loading control, in the presence or absence of BDNF and the presence or absence of short hairpin targeting expression of *BBS4* (sh*BBS4*). (B) Average activation of TRKB in hTERT-RPE1 cells quantified as the amount of pTRKB relative to the amount of TRKB for indicated treatments measured by ImageJ densitometry analysis. Error bars depict standard deviation across a minimum of 3 experiments. *significant change (p<0.01, t-test) from control; **significant change (p<0.01, t-test) from BDNF-treated control cells. (C) Western blot detection in hTERT-RPE1 cells of pTRKB and TRKB, as well as Actin, in the presence or absence of BDNF and the presence or absence of a short hairpin targeting the 3′UTR (sh*BBS4*) or a vector expressing *BBS4* (BBS4). (D) Quantification of the average activation of TRKB in hTERT-RPE1 cells quantified as the amount of pTRKB relative to the amount of TRKB for indicated treatments. *significant change (p<0.01, t-test) from control; **significant change (p<0.05, t-test) from BDNF-treated control. (E) Western blot detection SH-SY5Y cells pTRKB and TRKB in the presence of absence of BDNF and the presence or absence of sh*BBS4*. (F) Average activation of TRKB in SH-SY5 cells quantified as the amount of pTRKB relative to the amount of TRKB for indicated treatments. Error bars depict standard deviation across a minimum of 3 experiments. *significant change (p<0.01, t-test) from control; **significant change (p<0.01, t-test) from BDNF-treated control cells.

To explore a possible molecular link between BBS and BDNF, we first asked whether activation of TrkB is perturbed with loss of BBS4 in a ciliated cell line (hTERT-RPE1). hTERT-RPE1 cells are derived from retinal pigment epithelium, a site of active TRKB/BDNF signaling, and express *TRKB* endogenously ([20], [21]; Fig. S1A). We transfected cells with a short hairpin construct targeting *BBS4* (sh*BBS4*). By 48 hours after transfection of sh*BBS4* into hTERT-RPE1 cells, expression of *BBS4* mRNA and protein was effectively suppressed without disrupting endogenous expression of *TRKB* or *BDNF* (Fig. S1A–D). To examine the effect of BDNF on TrkB activation, cells transfected with either sh*BBS4* or empty vector were subsequently cultured in media supplemented with or without BDNF (50 ng/mL) for an additional 24 hours. Using antibodies to detect endogenous TRKB receptor or TRKB phosphorylated at tyrosine 705 (pTRKB; Sigma-Aldrich), we performed western blot analysis of whole cell lysates. A low level of endogenous pTRKB could be detected in control cells in the absence of added BDNF, likely due to low-level endogenous expression of *BDNF* ([21]; Fig. . The level of pTRKB relative to TRKB, however, was visibly enhanced with the addition of BDNF (Figure 1A). This correlated to a two-fold increase in receptor activation, calculated as the ratio of pTRKB protein to TRKB protein (Figure 1B). Phosphorylation of TRKB in *BBS4-*deficient cells treated with BDNF, however, was reduced by 53% to a level that was statistically indistinguishable from control cells cultured in BDNF-deficient media (Figure 1A–B). To verify that this defect was due to reduced BBS4 protein, we transfected cells with a second short hairpin construct targeted at the 3′UTR of the *BBS4* transcript. Upon transfection of this short hairpin construct we found that BBS4 protein levels were reduced (Fig. S1E). We also observed a significant decrease in the activation of TRKB in cells treated with BDNF (Fig. 1C–D). To determine if this could be rescued by *BBS4* expression, we co-transfected short hairpin-treated cells with a vector expressing the *BBS4* open reading frame without the 3′UTR such that it escapes short hairpin suppression. Upon co-transfection, BBS4 protein expression was rescued (Fig. S1E) as was activation of TRKB (Fig. 1C–D). The latter was significantly higher than in cells treated with shBBS4 alone (Fig. 1D).

To verify the potential relevance of our observations to neuronal signaling, we assessed the ability of BDNF to induce TRKB phosphorylation in a human neuroblastoma-derived cell line, SH-SY5Y. These cells express endogenous TRKB and respond to BDNF [22]. BBS4 protein expression was reduced upon treatment with short hairpin targeting it (Fig. S1F). We also detected a low-level of endogenous TRKB activation in control cells cultured in media without added BDNF, similar to hTERT-RPE1 cells (Figure 1E). The level of activation was significantly increased (2.2-fold) after addition of BDNF to cells (Figure 1E–F). Similar to hTERT-RPE1 cells, however, suppression of *BBS4* expression in these cells significantly reduced phosphorylation of the receptor, reducing activation to a level less than that in cells cultured in BDNF-deficient media (Figure 1E–F), providing further support for a role for BBS4 in activation of TRKB by BDNF.

### TRKB localization to cilia depends on BDNF and is disrupted by loss of BBS4

**Figure 2:**
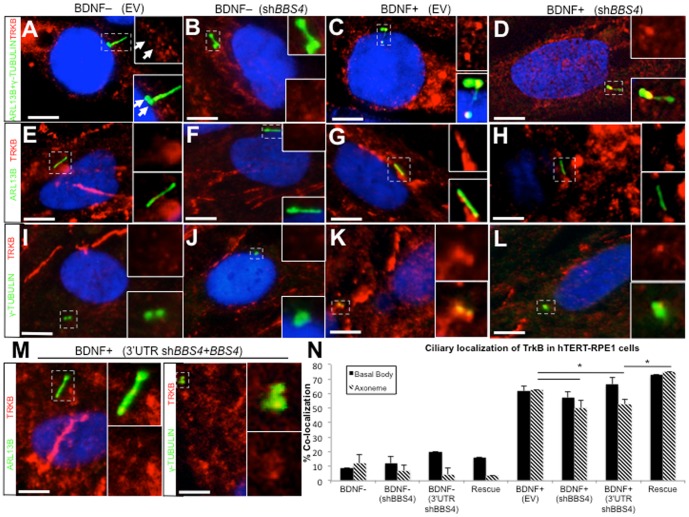
TRKB localizes to the basal body and axoneme of hTERT-RPE1 cells in the presence of BDNF. (A–M) Immunofluorescent staining of hTERT-RPE1 cells transfected with empty vector (EV), sh*BBS4* or both 3′UTR sh*BBS4* and *BBS4* expression construct (Rescue). Cells were either cultured in BDNF-deficient (BDNF-) or BDNF-supplemented (BDNF+) media and stained using antibody against TRKB (red) or ciliary markers labeling axoneme (ARL13B, green) and basal body (γ-tubulin, green, arrows). Region around cilia denoted by dashed box and magnified inset. Scale bar  = 10 µm. Imaged at 100× magnification. (N) Quantification of ciliary localization of TRKB calculated as the proportion of either basal bodies (black bars) or axonemes (striped bars) that co-localize with TRKB. Error bars represent standard deviation. *significant difference (p<0.01, chi-square test).

Many signaling pathways regulated by primary cilia are impeded when ciliary localization of their components is lost [23]. Furthermore, BBS4 is a member of the complex of proteins known as the BBSome, which can traffic cargo to cilia [8]. A role for *BBS4* in TRKB receptor activation by BDNF, therefore, might suggest that the receptor localizes to cilia. To investigate this possibility we labeled hTERT-RPE1 cells cultured in BDNF-deficient media with antibody against endogenous TRKB and with antibodies against markers of both the ciliary axoneme (ARL13B) and the centrioles (γ-tubulin), which include the basal body from which the axoneme extends. We labeled cells by double immunostaining with antibody against TRKB and both ciliary markers together to visualize total ciliary localization (Figure 2A–D). We also labeled cells with TRKB and ciliary markers individually to accurately assess localization to either the basal body or the axoneme (Figure 2E–L). Though TRKB could be detected throughout cells cultured without BDNF, we observed co-localization of TRKB with the ciliary axoneme in only a small proportion (11.9%) of empty vector-transfected (EV) control cells. Likewise, localization could only be detected in 8.5% of basal bodies (Figure 2A,E,I,M). Loss of BBS4 did not significantly alter localization to either structure (Figure 2B,F,J,N).

Ciilary localization of components of other pathways can be triggered in response to the presence of the ligand. For example, Shh effector, Smoothened, translocates to the cilium in the presence of Shh [24]. We, therefore, hypothesized that the presence of BDNF may influence localization of TRKB to the cilium. To test this, we added BDNF to the culture medium and co-immunostained control cells with antibodies against endogenous TRKB as well as ARL13B and γ-tubulin. After 24 hours of BDNF treatment we observed co-localization in 62.5% of axonemes and 61.9% of basal bodies indicating a significant increase in localization to both structures compared to cells cultured in BDNF-deficient media (Figure 2C,G,K,N). This localization was initiated shortly after BDNF treatment and persisted, as it could be detected in 90% of cells starting at 4 hours and continues at high levels at 8 and 12 hours of treatment (Fig. S2). Ciliary localization began to decrease at 24 hours, but was still present in a majority of cells 30 hours post-treatment (Fig. S2), indicating persistent localization of the receptor to the cilium in the presence of its ligand. To determine the importance of BBS4 in BDNF-dependent ciliary localization of TRKB, we examined sh*BBS4*-transfected cells treated with BDNF for 24 hours and immunostained for TRKB as well as ciliary markers. TRKB co-localization with basal bodies could be observed in 57.2%, a proportion that was not significantly different from control cells. However, a significantly smaller proportion of axonemes (50%) co-localized TRKB in sh*BBS4*-treated cells compared to control cells suggesting that axonemal localization is dependent on *BBS4* (Figure 2D,H,L,N). Suppression of *BBS4* with the second short hairpin targeting the 3′UTR also disrupted axonemal localization of TRKB, an effect that could be rescued by co-transfection with the *BBS4* expression construct (Figure 2M–N).

### Ciliary localization of activated TRKB is perturbed with loss of BBS4

**Figure 3:**
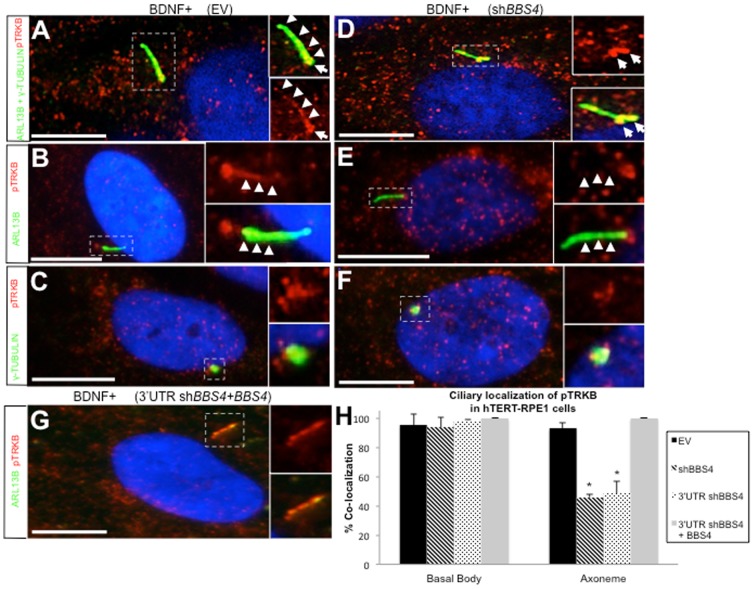
pTRKB in the ciliary axoneme is lost with depletion of *BBS4* expression. (A–G) Immunofluorescent staining of hTERT-RPE1 cells transfected with empty vector (EV), sh*BBS4* or both 3′UTR sh*BBS4* and *BBS4* expression construct. Cells were cultured in BDNF-supplemented media and stained using antibody against pTRKB (red) or ciliary markers labeling axoneme (ARL13B, green) or basal body (γ-tubulin, green). Region around cilia denoted by dashed box and magnified inset. Basal bodies highlighted by arrows and axoneme in (A,D) highlighted by arrowheads. Scale bar  = 10 µm. Imaged at 100× magnification. (H) Quantification of ciliary localization of pTRKB in transfected cells calculated as the proportion of either basal bodies or axonemes that co-localize with pTRKB. Error bars represent standard deviation. *significant difference (p<0.01, chi-square test).

The enhanced localization of TRKB to both the basal body and the axoneme in the presence of BDNF suggests that ciliary localization may be important for activation of the receptor. To explore this possibility, we investigated the localization of phosphorylated TRKB (pTRKB) in cells by immunostaining. In the absence of exogenous BDNF, pTRKB could be detected at very low levels in hTERT-RPE1 cells and ciliary localization could not be clearly discerned (Fig. S3). After addition of exogenous BDNF to the culture medium for 24 hours, pTRKB expression was more abundant and clearly visible by immunostaining (Figure 3). In BDNF-treated cells, we observed pTRKB localization to the axoneme in 93% of cells (Figure 3A,B,H). The activated receptor could also be detected at basal bodies in 95% of cells (Figure 3A,C,H). To determine if localization of pTRKB is altered with loss of *BBS4*, we assessed the ciliary localization of activated receptor in cells treated with either *BBS4* short hairpin. Though pTRKB localization to basal bodies was maintained in 93% and 98% of these cells, respectively, differences statistically insignificant from controls, localization at the ciliary axoneme could only be detected in 45.7% or 48.2% of cells, respectively (Figure 3D–F,H). This loss of localization was not due to loss of ciliogenesis since axonemes were clearly present in these cells (Figure 3E), though they were significantly shorter (Fig. S4A). To verify the specificity of this defect to loss of *BBS4*, we co-transfected 3′UTR *shBBS4-*treated cells with vector expressing *BBS4*. Basal body localization of pTRKB was unchanged, but localization could be observed in 100% of axonemes, consistent with a full rescue of the short hairpin phenotype (Figure 3G–H). Taken together, these observations suggest that, similar to TRKB, pTRKB localization to the ciliary axoneme, but not the basal body, is dependent on *BBS4* expression.

### Loss of KIF3A reduces TRKB activation without reduced basal body localization

**Figure 4:**
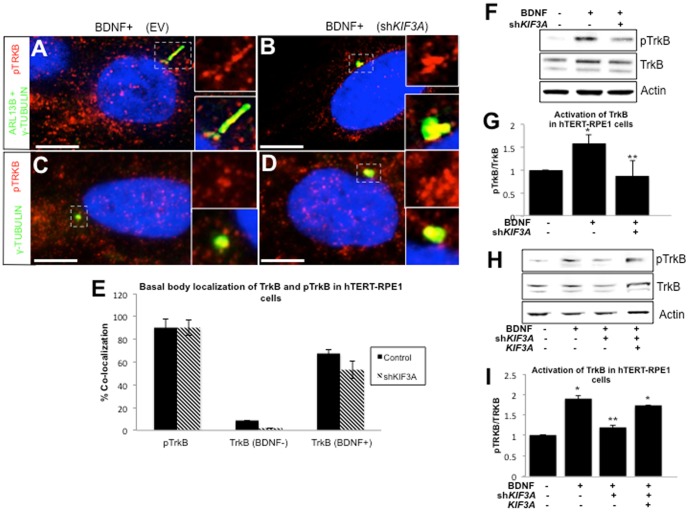
Reduced TRKB activation with loss of ciliary axoneme. (A–D) Immunofluorescent staining of empty vector (EV) control or sh*KIF3A-*treated hTERT-RPE1 cells cultured in BDNF-supplemented media and stained using antibody against pTRKB (red) or ciliary markers labeling axoneme (ARL13B, green) or basal body (γ-tubulin, green). Region around cilia denoted by dashed box and magnified inset. Scale bar  = 10 µm. Imaged at 100× magnification. (E) Quantification of basal body localization of pTRKB and TRKB in control (black) or sh*KIF3A*-treated (striped) cells calculated as the proportion that co-localize with pTRKB or TRKB with or without BDNF. Error bars represent standard deviation. No significant difference between control and sh*KIF3A*. (F) Western blot detection for pTRKB and TRKB in hTERT-RPE1 cells treated with or without BDNF and with or without sh*KIF3A*. (G) Quantification of TRKB activation calculated as the average ratio of pTRKB to TRKB protein, measured by ImageJ densitometry analysis. Error bars depict standard deviation across a minimum of three experiments. *significant change (p<0.01, t-test) from control; **significant change (p<0.01, t-test) from BDNF-treated control cells. (H) Western blot detection in hTERT-RPE1 cells of pTRKB and TRKB, as well as Actin, in the presence or absence of BDNF and the presence or absence of a short hairpin targeting the 3′UTR (sh*BBS4*) or a vector expressing *BBS4*. (I) Quantification of the average activation of TRKB in hTERT-RPE1 cells quantified as the amount of pTRKB relative to the amount of TRKB for indicated treatments. *significant change (p<0.01, t-test) from control; **significant change (p<0.05, t-test) from BDNF-treated control.

The reduced TRKB activation in sh*BBS4-*treated cells coupled with the loss of TRKB and pTRKB localization from ciliary axonemes suggests that axonemal localization may be associated with proper activation. To investigate this possibility, we obtained a short hairpin construct against *KIF3A* which is necessary for production of a ciliary axoneme, but not centrioles [25]. Short hairpin targeting *KIF3A* (sh*KIF3A*) reduced the expression of *KIF3A* by 61% by 48 hours post-transfection (Fig. S4B). We performed immunofluorescent staining of BDNF-treated hTERT-RPE1 cells to determine the intracellular localization of pTRKB in relation to the ciliary axoneme (anti-ARL13B) and basal body (anti-γ-tubulin). Consistent with previous reports, cells depleted of *KIF3A* expression exhibited a loss of ciliogenesis in 80% of cells compared to empty vector-transfected control cells, evidenced by a loss of ARL13B staining throughout the axoneme ([25], [26]; Figure 4A–B). Immunofluorescence could be detected at a single structure in each cell, however, potentially representing the centrioles labeled by γ-tubulin (Figure 4B). To confirm this, we labeled cells with pTRKB and γ-tubulin alone (Figure 4C–D). In spite of impaired axonemal extension in these cells, co-localization with γ-tubulin of either TRKB or pTRKB was not significantly altered in comparison to control cells (Figure 4E). Furthermore, addition of BDNF to cells significantly increased basal body localization of TRKB in both control and sh*KIF3A-*treated cells (Figure 4E). We next asked whether the loss of *KIF3A* would alter TRKB activation by assessing the protein levels of both TRKB and pTRKB in whole cell lysates of sh*KIF3A-*treated cells treated with BDNF. Compared to control cells treated with BDNF, the ratio of pTRKB to TRKB was reduced by 38% in sh*KIF3A* cells, representing a significant decrease in activation of TRKB (Figure 4F–G). We further validated these observations by targeting *KIF3A* with a second short hairpin directed at the 3′UTR of the transcript. Consistently, treatment of these cells significantly impeded activation of TRKB by BDNF (Figure 4H–I). This was rescued by co-transfection of cells with a *KIF3A* expression construct (Figure 4H–I).

## Discussion

Here we report a role for the Bardet-Biedl Syndrome gene, *BBS4*, in regulation of BDNF signaling through the TRKB receptor in cultured human cells. Upon loss of *BBS4* expression, TRKB activation by BDNF was significantly reduced. In addition, we observed localization of TRK to cilia specifically in the presence of BDNF. Loss of *BBS4* perturbed localization of TRKB as well as its phosphorylated form, pTRKB, at the ciliary axoneme, but not basal bodies. Finally, ablation of *KIF3A* expression impaired axonemal extension and also reduced activation of TRKB by BDNF. Taken together, these findings implicate a ciliopathy gene, *BBS4*, in the regulation of BDNF signaling through TRKB and suggest its importance in localization of the receptor to the axoneme of primary cilia.

These results offer novel insight into the intracellular regulation of BDNF signaling, a complex pathway that is likely regulated by a number of mechanisms. This study suggests that regulation of the pathway may be dependent on primary cilia. Specifically, we demonstrate the necessity of proteins associated with trafficking of ciliary cargo (BBS4) or with ciliogenesis (KIF3A) in activation of TrkB by BDNF. Importantly, these observations are potentially consistent with previous reports of intracellular regulation of BDNF-dependent activation of TrkB. For example, in the plasma membrane of neurons the presence of BDNF can trigger the translocation of TrkB receptor to lipid rafts from non-raft regions, potentially enhancing the ability of the receptor to signal by placing it in an environment enriched with signaling molecules [27]. There is evidence to suggest that primary cilia in epithelial cells are enriched for lipid rafts [28], supporting our observation that the presence of BDNF triggers localization of TRKB to the cilium. This might suggest the possibility that the localization of TrkB to the cilium in the presence of BDNF is important for placement of the receptor in an environment rich with other effector molecules that allow for proper transduction of the pathway. In addition, clathrin-mediated endocytosis of activated Trk receptors is necessary for some aspects of ligand-mediated signaling and BDNF-TrkB binding triggers endocytosis in hippocampal neurons [29]. Recent evidence has revealed the base of the primary cilium to be an active site of clathrin-mediated endocytosis [30], offering an additional potential mechanistic link. It is possible that transport of the receptor to the endocytosis-rich region near cilia by BBS proteins is necessary for proper endocytic trafficking and associated signaling by BDNF through TrkB. It is also likely that BDNF signaling is regulated at other sites in the cell as well as through the production of various isoforms of both the receptor and the ligand. Given the importance of BDNF signaling through TRKB in obesity and other neuronal phenotypes, understanding its regulation at distinct intracellular sites will be necessary to understand how disruption of localization and interaction with cellular components contributes to its dysfunction and, potentially, to disease phenotypes.

It is important to note that our investigation is limited to assessment of TrkB activation by BDNF in an *in vitro* system of cultured cells. The potential relevance to disease phenotypes associated with BBS or other ciliopathies remains to be determined. If perturbation of TrkB activation *in vivo* in animal models of ciliopathies is observed, consistent with our observations in cells, this would support BDNF signaling as a possible mechanism for traits associated with these diseases. This might include obesity. The hyperphagic childhood obesity associated with loss of BDNF expression or loss of TrkB is highly reminiscent of that seen in BBS and other obesity ciliopathies [19], [31]. Given that evidence implicating a direct causal role for other anorexigenic signals, such as leptin, is conflicting [32], [33], it is possible that perturbation of satiety signals or of neurogenesis induced by BDNF in the hypothalamus may offer an alternate or additional explanation for the increased food intake and weight gain in BBS. Much attention has focused on the putative role of CART/POMC neurons in the arcuate nucleus of the hypothalamus, for example, but there is little evidence to suggest a role for BDNF in the activity of these neurons [14], [34]. Therefore, it is possible that perturbed ciliary function may disrupt BDNF signaling in other hypothalamic populations involved in food intake such as the melanocortin 4 receptor (MC4R) expressing neurons in the ventromedial hypothalamus. Finally, it will also be necessary to consider the role of BDNF as a neurotrophin in the development and patterning of the hypothalamus. This will require investigation of the production of specific neuronal populations in the absence of ciliopathy proteins and determining what associated deficits, if any, may be dependent on proper embryonic BDNF signaling through TrkB.

Taken together, our findings offer novel preliminary insight into the intracellular regulation of BDNF signaling through TrkB at the primary cilium. The implications of this disruption may extend to a spectrum of neuronal phenotypes. Further studies investigating ablation of cilia in specific areas of the brain, and what effects that has on BDNF, will be necessary to elucidate how this disruption may underlie associated phenotypes.

## Supporting Information

Figure S1 **mRNA and protein expression in sh** ***BBS4-*** **transfected cells.** (A–B) Quantitative RT-PCR detection of *TRKB* (A) or *BBS4* (B) in hTERT-RPE1 cells showing expression relative to GAPDH in empty vector (control) cells or sh*BBS4* cells 48 hours post-transfection. (C) Detection of BBS4 protein by western blot in hTERT-RPE1 cells transfected with or without sh*BBS4*. (D) Quantitative RT-PCR detection of *BDNF* in hTERT-RPE1 cells showing expression relative to GAPDH in control or sh*BBS4* cells. (E) Detection of BBS4 protein in cell transfected with pLKO.1 empty vector, short-hairpin targeting 3′UTR (pLKO.1-sh*BBS4*), pCS2 empty vector, or BBS4 expression vector (pCS2-*BBS4*). (F) Detection of BBS4 protein by western blot in SH-SY5Y cells transfected with or without sh*BBS4*. Error bars represent standard deviation across a minimum of three experiments.
(TIFF)

Figure S2 **Time course of BDNF and ciliary localization of pTRKB in hTERT-RPE1 cells.** Quantification of proportion of untransfected hTERT-RPE1 cells exhibiting ciliary (basal body and axoneme) localization after treatment with BDNF added to culture media for the indicated lengths of time.
(TIFF)

Figure S3 **pTRKB in the absence of BDNF.** (A–C) hTERT-RPE1 cells cultured without addition of BDNF immunostained with antibodies against ARL13B+γ-tubulin (green) and pTRKB (red).
(TIFF)

Figure S4 **Reduced ciliary length and targeting of** ***KIF3A*** **in hTERT-RPE1 cells.** (A) Quantification of average length of cilia in sh*BBS4*-treated cells. *significant change from control, p = 0.03, t-test. (B) Quantitative RT-PCR detection of *KIF3A* in empty vector (control) cells or sh*KIF3A*-transfected cells shown as expression relative to that in control using GAPDH as reference. Error bars represent standard deviation across a minimum of three experiments.
(TIFF)
