# Supplementary figures and images for: BBS4 Is Necessary for Ciliary Localization of TrkB Receptor and Activation by BDNF
Source: PLoS One. 2014 May 27;9(5):e98687. doi: 10.1371/journal.pone.0098687 (PMC4035337; doi:10.1371/journal.pone.0098687)

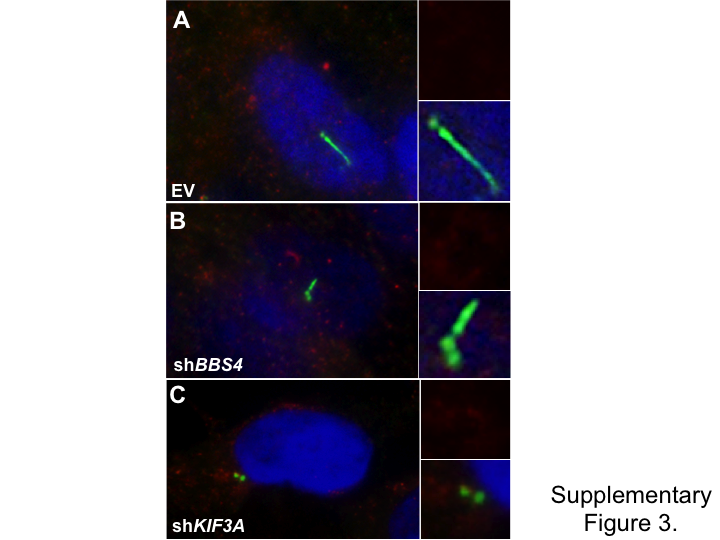

Supplement: Figure S3 — pTRKB in the absence of BDNF. (A–C) hTERT-RPE1 cells cultured without addition of BDNF immunostained with antibodies against ARL13B+γ-tubulin (green) and pTRKB (red). (TIFF) [file pone.0098687.s003.tiff]
